# Modeling Inflammation in Autism Spectrum Disorders Using Stem Cells

**DOI:** 10.3389/fped.2018.00394

**Published:** 2018-12-12

**Authors:** Beatriz C. Freitas, Arianna Mei, Ana Paula D. Mendes, Patricia C. B. Beltrão-Braga, Maria Carolina Marchetto

**Affiliations:** ^1^Laboratory of Disease Modeling, Department of Microbiology, Institute of Biomedical Sciences, University of São Paulo, São Paulo, Brazil; ^2^Laboratory of Genetics, The Salk Institute, La Jolla, CA, United States; ^3^School of Arts, Sciences and Humanities, University of São Paulo, São Paulo, Brazil

**Keywords:** autism spectrum disorder (ASD), disease modeling, neuroinflammation, neurodevelopmental disorders, induced pluripotent stem (iPS) cells

## Abstract

Recent reports show an increase in the incidence of Autism Spectrum Disorders (ASD) to 1 in every 59 children up to 8 years old in 11 states in North America. Induced pluripotent stem cell (iPSC) technology offers a groundbreaking platform for the study of polygenic neurodevelopmental disorders in live cells. Robust inflammation states and immune system dysfunctions are associated with ASD and several cell types participate on triggering and sustaining these processes. In this review, we will examine the contribution of neuroinflammation to the development of autistic features and discuss potential therapeutic approaches. We will review the available tools, emphasizing stem cell modeling as a technology to investigate the various molecular pathways and different cell types involved in the process of neuroinflammation in ASD.

## Introduction

Autism Spectrum Disorder (ASD) is a lifelong neurodevelopmental disorder characterized by the impairment of social abilities and cognitive functions that can manifest cortical disorganization or neuroinflammation states (1). As a complex polygenic disorder with several degrees of severity, the causes of ASD are not completely elucidated. Autism can be of unknown gene etiology (idiopathic) (2) or a specific syndromic disorder, such as Rett Syndrome or Fragile X where single gene mutations can manifest ASD features. Elucidating the etiology of ASD could help understanding the increase in incidence (3) and drive research efforts toward novel therapeutic approaches.

Earlier recognition of ASD symptoms results in better patient prognosis after behavioral therapy (4). Cortical changes, such as increased head circumference and brain volume reported for a subset of ASD toddlers, allow for potential clinical diagnosis before 1 year of age, especially when aligned with a genetic investigation of known genes that play a role in neural development (4). Sequencing of whole genomes of families where the proband was affected with ASD uncovered rare *de novo* mutations on genes that segregate with ASD (5, 6). These mutations are heterogeneous and only account for < 1% of ASD cases (7), highlighting the polygenic nature of ASD. A subset of the genes identified in ASD (*FMR1, CHD8, DYRK1, NLGN3, PTEN*) and 16p11 deletion, are likely involved in brain volume changes and neural connectivity, and its particular function can be studied in animal models and genetically engineered human cells (8). The majority of idiopathic ASD cases lack a known culprit gene(s), with phenotypes ranging across a wide pathological spectrum. In those cases, differential scientific approaches, such as the use of stem cell modeling and comprehensive *in silico* data analysis are required. Furthermore, twin studies suggest that hereditary mechanisms and environment also play a role in ASD during mid-fetal brain development (9).

Several studies report immune system misbalances in ASD (10–12). Particularly, immune system involvement has been associated with three major circumstances: 1-*specific immune-related genes*, 2-*maternal risk factor* (ex. gestational diabetes and/or obesity), and 3-*maternal immune activation (MIA) during pregnancy* (ex. viral infection) (13). A common feature associated with these three scenarios is the altered number of synapses in the fetus leading to deficits during neurodevelopment.

Global expression differences in genes related to inflammatory response in ASD individuals were reported by several studies (14–16). Transcriptome analysis of ASD post-mortem brains revealed a close association between ASD and the genes related to glial cell activation and genes belonging to immune and inflammatory categories (17). The *MET* gene (proto-oncogene receptor, tyrosine kinase) known to regulate cell immune functions amongst other roles (18) is the immune gene most closely associated with ASD (19), coding for a receptor that binds to hepatocyte growth factor (HGF). Single nucleotide polymorphisms (SNPs) were described at high incidence in ASD and reduced expression of aberrant *MET* was shown in post-mortem brains of ASD individuals (20). SNPs generating deficiencies of the complement component 4 (C4) gene function, located in the MHC region, have also been associated with ASD (21, 22). Additional components of the complement cascade (*C3, Masp1, Masp2, C3aR*, and *C5aR*) participate in microglial function and neuronal migration and are potentially implicated in altered cortical development in ASD (23, 24).

Maternal history of increased inflammation, such as development of eczema, psoriasis, and asthma was correlated with a 20–40% increased risk for both ASD and other developmental disorders (25). Other risk factors apart from the immune deficiencies mentioned are gestational diabetes, obesity, and aging that were linked to an increased chance of a child to developing ASD (26, 27). Lastly, acquiring MIA due to a viral infection during pregnancy shows evidence of an additional increased risk for developing ASD (28).

Anomalies in immune signaling pathways are reported for ASD (29). Specifically, altered cytokine profiles and immunoglobulin G (IgG) levels were associated with intensified cellular immunity and general amplified neuroinflammation. Detection of misbalanced expression of inflammatory cytokines (IL-6, TNFα, TGFβ, IL-17, and IL-2) was reported in ASD patients' blood (30). Unfortunately, due to the complexity of this disorder, none of these alterations resulted in a biomarker panel for predicting ASD prognosis. In this review we will highlight the role of inflammation in ASD focusing on the data obtained from stem cell modeling.

## Stem Cell Models in Autism Spectrum Disorder (ASD)

**Figure 1:**
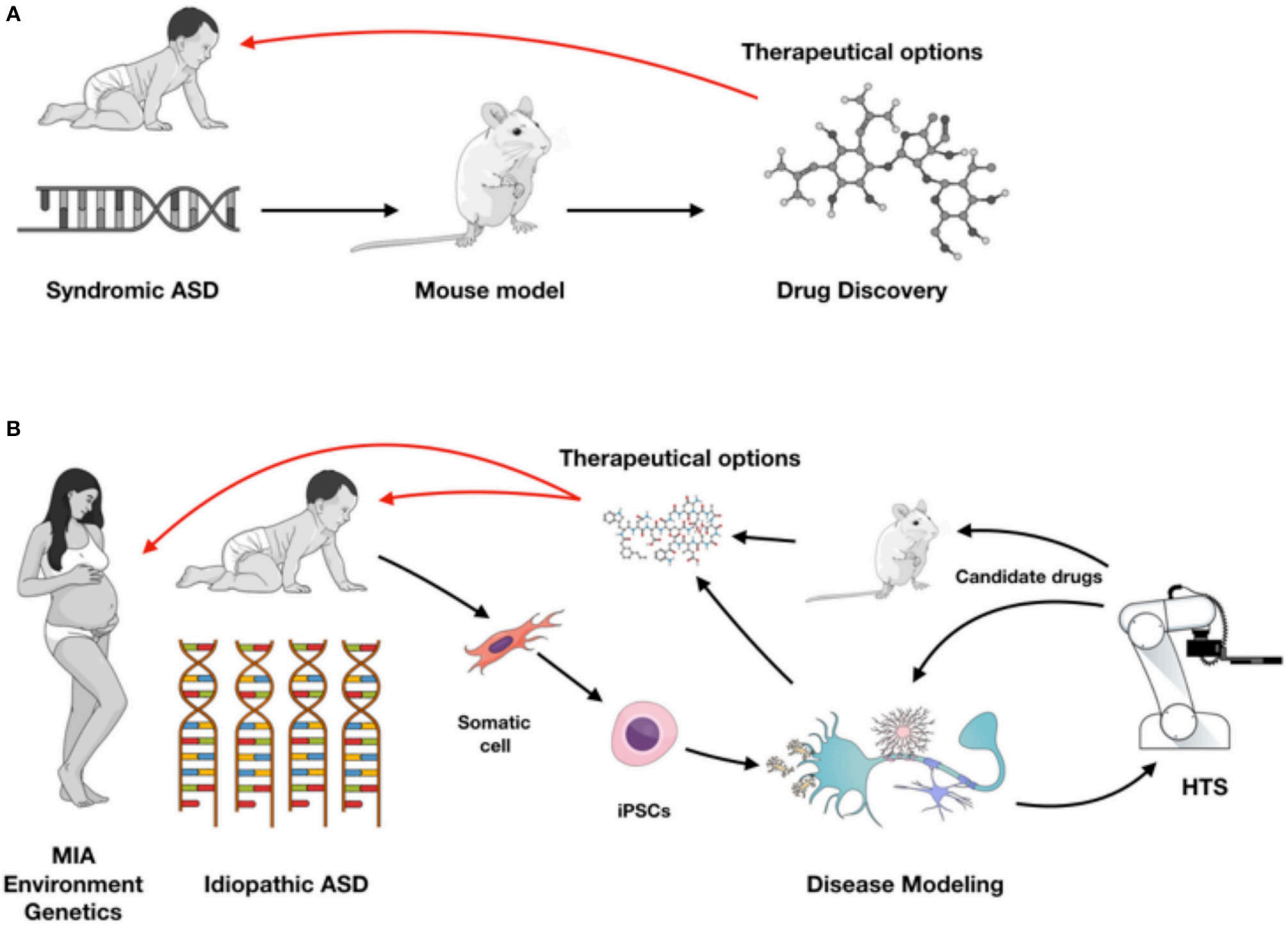
**(A)** Syndromic ASD historically has been studied using rodent models leading to drug discovery for therapeutic approaches (red arrow). **(B)** With iPSC modeling both syndromic and idiopathic ASD forms can be investigated in different cell types from the nervous system, or in a complex co-culture model system and used for high-throughput screening (HTS). Validation of target compounds can be performed on iPSC-derived and rodent models, providing therapeutic possibilities as early as during brain development (red arrows). Figure created in the *Mind the Graph* platform with subscription purchased by the authors.

Neurodevelopmental polygenic disorders, such as ASD are notably attractive for the application of stem cell technology as early hallmarks of neuronal development can be dissected *in vitro* and patients' unique genetic background can be recapitulated (31). Embryonic stem cells (ESCs) and induced pluripotent stem cells (iPSCs) have the ability to generate functional human neuronal and glial cells *in vitro*, and can self-organize into 3D brain organoid cultures (32) (Figure 1). iPSCs recapitulate aspects of neuronal development process while preserving the genetic background from ASD patients and thus are more frequently used to model idiopathic conditions. Additionally, isogenic lines can be developed to understand the impact of a specific mutation in different genetic backgrounds. While gene-editing technology is ideal for understanding the function of a single gene, the results are not always generalizable to the autistic condition. In that case, having iPSC lines from a cohort of patients with a specific endophenotype (ex. macrocephaly) can be informative for understanding ASD *in vitro* phenotypes. Unfortunately for idiopathic polygenic ASD, isogenic lines are not always a viable option.

## Idiopathic ASD

Despite being a polygenic disorder, there appears to be converging phenotypes in ASD. A common finding from ASD studies reporting the generation of iPSC-derived neurons from syndromic or idiopathic ASD is *altered synaptic activity* (33–41). iPSC technology allows for the interrogation of the specific neurobiological foundations underlying common synaptic defects and synaptopathy in the context of neurodevelopmental disorders such ASD (42).

Aiming to recapitulate clinical post-mortem findings in neurons, one group using gene expression analyses on three-dimensional cultures derived from iPSCs showed up-regulation of genes involved in cell proliferation, neuronal differentiation, and synaptic formation (34). In this study, Mariani et al. (34) suggested that overexpression of the gene *FOXG1* can generate an overproduction of GABAergic neurons, which can cause increase of brain volume and imbalance of excitation and inhibition systems in the developing cortex in ASD.

Increased proliferation of iPSC-derived neural progenitors from ASD individuals with enlarged brains was also reported by Marchetto et al. (35). Importantly, the authors observed a significant correlation between ASD patients' brain volumes *in vivo* and cell proliferation rates *in vitro*, indicating that iPSC-derived neural cells can be used as a proxy to infer specific patients' metrics (i.e., brain volume). Marchetto et al. (35) also showed a significant reduction in the density of excitatory synapses in ASD iPSC-derived neurons and decreased neuronal bursting. To date, these findings suggest that despite the overgrowth of the brain size in these young children, their neurons are immature or unhealthy.

Though neuronal phenotypes have been explored in more depth in ASD; glial cells play an important role in the disease as well. A recent study using iPSC-derived neurons and iPSC-derived astrocytes from idiopathic ASD individuals demonstrated that ASD astrocytes influence neuronal morphology and function, evidenced by co-culture experiments (43). Remarkably, when astrocytes from neurotypical individuals were co-cultured with ASD neurons they improved morphological and functional features that were not observed when ASD neurons were co-cultured with ASD astrocytes. The researchers identified interleukin-6 (IL-6) as a key cytokine responsible for the effect on neuronal activity by showing synaptogenesis recovery in ASD neurons after blocking IL-6 release in ASD astrocytes. The researchers also hypothesized that an increased expression of IL-6 in ASD astrocytes might be one of the triggers of the observed neuronal defects (43). Interestingly, elevated levels of IL-6, along with other inflammatory cytokines, were reported in the brains and blood of autistic subjects and IL-6 has been found to be a key mediator in MIA models (30, 44, 45).

## Inflammation and Syndromic ASD

### RETT Syndrome (RTT)

RTT is a monogenetic progressive neurologic disorder that shares proposed mechanistic and core symptoms with ASD, with mutations in the X-linked gene *MeCP2* occurring in ~90% of patients with RTT (46–48). RTT patients are predominantly female, as affected male patients are rare, and those who survive usually die at early ages (49–51). RTT patients have progressive neurological abnormalities starting at 6–18 months after birth. Following a period of progressive neurological damage there is deceleration of head growth, severe motor abnormalities, and loss of language skills. Along with the RTT onset of development stagnation comes impaired motor function, stereotypic hand wringing, hypotonia, seizures, autistic behavior, encephalopathy, and respiratory dysfunction (46, 49). Amir et al. identified mutations in methyl CpG binding protein 2 (*MeCP2*) as the primary genetic cause of RTT (47) and Chahrour et al. showed that MeCP2 binding can both repress and activate transcription (52).

Reduced MeCP2 expression is also found in forebrain post-mortem tissue from idiopathic ASD individuals, suggesting similar epigenetic dysregulation in some cases of idiopathic ASD and RTT (48). Human iPSC-derived neural cells from RTT patients show fewer synapses, reduced soma size and dendritic spine density, altered calcium signaling and decreased spontaneous excitatory post-synaptic currents (33, 46). Marchetto et al. showed rescue in synapses phenotype of RTT after treatment with IGF-1 (Insulin Growth Factor Like-1) compound, which is now in clinical trials for RTT (33). Increased dosage of MeCP2 protein also results in severe neurodevelopmental delay with epilepsy and autistic behavior. Interestingly, iPSC-derived neurons from patients with the *MECP2* duplication syndrome show increased synaptogenesis and dendritic complexity resulting on abnormal network synchronization compared to controls (37). These results indicate that *MECP2* gene dosage is critical for proper human neurodevelopment.

Neurons were thought to be the most relevant cell type for RTT pathology due to the high expression levels of MeCP2 protein in these cells. Based on previous data showing the non-cell autonomous effect in rodents (53), a group in 2014 was able to confirm the negative effect of MeCP2-mutant astrocytes in neuronal morphology using iPSCs. Furthermore, IGF-1 and GPE (IGF-1 cleaved peptide) was used to partially rescue the neuronal deficits caused by mutant astrocytes (54). To date, the direct role of RTT-derived astrocytes in inflammation has not been extensively explored.

Several studies demonstrated that the immune system is involved in RTT in early life. Microglia activation and/or proliferation and defective BDNF signaling is described in RTT (55). Maezawa et al. proposed that RTT resting microglia are sensitive to both immunological stimuli and neuronal/astrocytic signals causing neuroinflammation and, consequently, affecting brain development (56, 57).

### Cyclin-Dependent Kinase-Like 5 Disorder (CDKL-5)

CDKL-5 is a kinase protein highly expressed in neurons, but its specific function inside the cell is poorly known (58). Mutations in X-linked *CDKL5* gene have been identified in the early onset seizures variant of RTT and male X-linked epileptic encephalopathy. CDKL5 expression is present in neurons suggesting an important role in neuronal development. In certain brain regions, MeCP2 protein can regulate the expression of *CDKL5* gene and in turn CDKL5 can phosphorylate MeCP2. Downregulation of CDKL5 expression inhibits neurite outgrowth and maturation of the neuronal network in rodents (59). iPSC-derived neurons from patients with CDKL5 mutations exhibit aberrant dendritic spines, suggesting a common function of CDKL5 in mice and humans (58, 60).

The immune system misbalance in Rett syndrome (RTT) can be explained in part by impaired regulation of the inflammatory system. In rodent models MeCP2 regulates gene expression in microglia and macrophage (61). Leoncini et al. (62) showed that cytokine levels of RTT patients are misregulated in the blood and that there are differences in cytokine profiling depending on the RTT mutation (*MECP2* vs. *CDKL5*). The researchers also pointed out that omega-3 polyunsaturated fatty acids (PUFAs) partially counterbalanced the cytokine changes and the aberrant redox homeostasis and ameliorated the inflammatory status (62).

### Fragile X Syndrome (FXS)

Patients with FXS show intellectual disability, hyperactivity and a range of autistic-like behaviors. FXS is characterized by the absence of expression of the fragile X mental retardation 1 (*FMR1*) gene due to CGG trinucleotide repeat expansion in the 5′ untranslated region of *FMR1*. This aberrant expression leads to hypermethylation and gene silencing, leading to the decrease or absence of FMRP (fragile X mental retardation protein) (46, 63).

FMRP is a selective RNA-binding protein that regulates the translation of many genes at synaptic sites. It was shown that the lack of FMRP leads to the excess and dysregulation of mRNAs translation, which leads to alterations in the synapse function, increased numbers of spines and neuronal overexcitability (64). A recent study using a Drosophila Fragile-X model showed phagocytosis defects in innate immune cells (65). Loss of *FMR1* also causes aberrant differentiation in human iPSC-derived neural progenitor cells, particularly a marked induction of the astrocyte marker glial fibrillary acidic protein (GFAP) (66). In parallel, research also shows increased astrocyte activation in *FMR1* knockout mouse cerebellum (67), indicating a possible increase in inflammation on both human and mouse models. Although cytokine imbalance was seen with increased IL-6 levels in mouse models (68), no iPSC-derived study has recapitulated these glial findings *in vitro*. Using ASD and Fragile-X iPSC-derived neurons, a study compared the molecular pathways between these two disorders by global gene expression and DNA methylation profiling, and identified several cytokine molecules, BMP7, and HLA-related abnormalities with aberrant FMRP1 expression (69).

## Animal Models used for ASD

Mouse modeling is a crucial tool for autism spectrum studies with the generation of genetically modified strains focusing on understanding the basic features of abnormal neurodevelopment in ASD.

A relevant environmental risk factor known to recapitulate aspects of ASD pathology was extensively studied by Patterson and his collaborators (70). His group induced an acute inflammatory response on expectant pregnant rodents, causing maternal immune activation (MIA) leading to autistic phenotypes in the offspring (70). The mechanism for the development of autistic traits was later correlated with the release of IL-6, amongst other cytokine misbalances especially in CD4^+^ T cells. The offspring from MIA models displayed general alteration of their immune profile and function, and interestingly, bone marrow transplantation of MIA-derived mice into irradiated control mice did not recapitulate the MIA phenotypes illustrating the importance of the peripheral system context in the immune system activation (71). Using CRE-induced models for local depletion of IL-6 receptors, Patterson's group showed that the placental IL-6 signaling is directly involved in fetal brain development and behavior and when IL-6 receptors were depleted in the placenta the MIA model had no effect on the offspring (72). Recent results from Cho et al. (73) also implicate the maternal interleukin-17a pathway in mice promoting autism-like phenotypes in the offspring. Their data suggest that therapeutic targeting of Th17 cells in susceptible pregnant mothers could potentially reduce the likelihood of bearing children with inflammation-induced ASD-like phenotypes (73).

Another relevant study proposed the hypothesis that blood marrow transplantations could improve the phenotype of mice with syndromic forms of ASD, such as RTT (74). The concept was based on the idea that healthy microglia would rescue RTT phenotypes in mouse models. While this is an appealing concept, several groups attempted to repeat the experiment using similar methodologies and the results have not been confirmed (75). Although microglia was not necessarily the culprit in the rescue of MeCP2-deficient phenotype, it is anticipated that this cell type plays an important role in the disease, particularly given its ability for synaptic pruning (76). Future studies will be crucial to consider the potential use of microglia replacement as a therapeutic strategy for ASD.

Mouse models lacking microglial fracktalkine receptor gene (*CX3CR1*) displayed increased microglial IL-1β resulting in an enhanced tauopathy, immature synapses throughout the cortex and connectivity imbalance with ASD-like behaviors (77). Blocking IL-1β/p38 MAPK pathways significantly improved the mouse phenotype and could be a promising therapeutic option (78). Interestingly, mouse models for RTT using MeCP2 KO mice crossed with mice with *CX3CR1* mutations recovered the MeCP2 deficient phenotype, suggesting that blocking the fracktalkine receptor is a possible therapeutic approach for RTT (79).

In 2011, a group led by Gail Mandel demonstrated the role for glia in the progression of RTT (53). Using MeCP2 KO animals and astrocyte-specific GFAP promoter controlling MeCP2 expression, they showed that astrocyte depletion of MeCP2 was detrimental to neurons in a non-cell autonomous fashion. Interestingly, the re-expression of MeCP2 in GFAP-driven cells was sufficient to rescue the RTT phenotype, and systemic delivery of MeCP2 was used as a therapeutic approach for female mouse models (80). Recent work demonstrated that MeCP2 expression in astrocytes affects glutamatergic synapses, highlighting the importance of astrocytes to ensure proper synaptic connectivity (81). Using primary rodent cells another group found that MeCP2-mutant astrocytes had an exaggerated inflammatory and oxidative stress response to LPS (Lipopolysaccharide) stimulation when compared to control cells, suggesting anti-inflammatory approaches for disease treatment (82).

## Limitations of Current Models

All current *in vivo* and *in vitro* models have increased our overall mechanistic knowledge of ASD, however the complexity of idiopathic autism and the influence of epigenetic and environmental factors poses a challenge to modeling non-syndromic forms using rodent models. Furthermore, when attempting to study aspects of neuroinflammation, where specific cytokines can play a crucial role in the disorder and are species-specific (83), humanized models can be instrumental to obtain therapeutically relevant information. As iPSCs emerge as an important *in vitro* model for translational research, current limitations include the restricted complexity for cytokine signaling and the absence of body systemic interaction. Future improvements to iPSC technology will incorporate circulating blood and mature cell types to the current model systems.

## Final Remarks

Increasing evidence supports the idea of immune system activation in ASD. iPSCs are crucial for a better understanding of complex polygenic diseases with heritable and sporadic forms, such as ASD in order to develop novel biomarkers and identify targets for future therapeutics (35). Deriving brain cells from iPSCs can in fact provide a unique opportunity to explore and identify early phenotypes in autism in opposition to secondary phenotypes that are a downstream consequence of the pathology. Moreover, iPSC technology is particularly valuable over the two most used research approaches to study and understand brain biology and pathology, namely post-mortem samples and mouse models. Post-mortem tissue has inherent limitations for studying a neurodevelopmental disease, mainly because it cannot be manipulated to test experimental hypotheses and provides only a brief snapshot in disease progression (84). Animal models can be genetically and pharmacologically manipulated, however, when it comes to behavioral, affective and cognitive changes, animal models may not always recapitulate the human phenotype (84, 85). Ideally for a complex disorder, such as ASD one would take advantage of multiple models, using them as a base to build the background knowledge of the mechanism of the disorder, studying each individual player (i.e., neuron, glia) and as a complex niche (*in vivo*, in co-culture or brain organoid systems). The next step would be developing a high-throughput screening (HTS) system capable of identifying disease biomarkers and potentially detecting compounds that rescue a phenotype. Since ASD is a complex developmental disorder, studying iPSC-derived fetal-like cells is ideal for establishing a therapeutic approach as early as during gestation time (Figure 1). Recent research indicates a relevant role for the gut microbiota in autism (86), specially regarding inflammatory components (87–89). It would be interesting to see upcoming technologies, such as “organs-on-a-chip” that can integrate brain development with gut signaling to an *in vitro* platform.

iPSC technology is also a remarkable scientific tool to test compounds that could be used in the clinic for ASD individuals. The grouth factor IGF1 was first tested in RTT mouse models (90) and then iPSC from ASD patients (33, 35) and it is now in clinical trials for syndromic and idiopathic forms of ASD^1^^,^ ^2^^,^ ^3^^,^ ^4^^,^ ^5^ (91, 92). The compound Luteolin (93) attenuates IL-6-mediated astrogliosis in human iPSC-derived neural aggregates and was proposed as a candidate preventive substance for maternal immune activation-induced abnormalities (94). Remarkably, Luteolin dietary intake improves ASD symptoms and reduces serum levels of TNF and IL-6 (95).

In summary, iPSC technology is an invaluable tool for asking questions about human-specific neuroinflammation mechanisms, such as the ones observed in syndromic and non-syndromic forms of ASD. Importantly, iPSCs can generate a platform for the discovery of new anti-inflammatory compounds that are tailored to the ASD-specific genetic background and neuropathological phenotypes and offer potential therapeutic options for both onset and progression of the disorder.

## Author Contributions

All authors listed have made a substantial, direct and intellectual contribution to the work, and approved it for publication.

### Conflict of Interest Statement

The authors declare that the research was conducted in the absence of any commercial or financial relationships that could be construed as a potential conflict of interest.

## Footnotes

^1^*A Pilot Treatment Study of Insulin-Like Growth Factor-1 (IGF-1) in Autism Spectrum Disorder*. Available online at: https://ClinicalTrials.gov/show/NCT01970345

^2^*A Safety Study of NNZ-2566 in Patients With Fragile X Syndrome*. Available online at: https://ClinicalTrials.gov/show/NCT01894958

^3^*Treatment of Rett Syndrome With Recombinant Human IGF-1*. Available online at: https://ClinicalTrials.gov/show/NCT01777542

^4^*Clinical Trial in 22q13 Deletion Syndrome(Phelan-McDermid Syndrome)*. Available online at: https://ClinicalTrials.gov/show/NCT01525901

^5^*Treatment of Rett Syndrome With rhIGF-1 (Mecasermin rDNA Injection)*. Available online at: https://ClinicalTrials.gov/show/NCT01253317
